# Salidroside suppressing LPS‐induced myocardial injury by inhibiting ROS‐mediated PI3K/Akt/mTOR pathway *in vitro* and *in vivo*

**DOI:** 10.1111/jcmm.12871

**Published:** 2017-09-14

**Authors:** Lvyi Chen, Peng Liu, Xin Feng, Chunhua Ma

**Affiliations:** ^1^ School of Pharmacy South‐Central University for Nationalities Wuhan China; ^2^ Institute of Tibetan Medicine China Tibetology Research Center Beijing China; ^3^ Department of Physiology and Pharmacology China Pharmaceutical University Nanjing China

**Keywords:** salidroside, LPS, myocardial injury, ROS, H9C2, PI3K/Akt/mTOR

## Abstract

The purpose of the present study was to investigate the effect of salidroside (Sal) on myocardial injury in lipopolysaccharide (LPS)‐induced endotoxemic *in vitro* and *in vivo*. SD rats were randomly divided into five groups: control group, LPS group (15 mg/kg), LPS plus dexamethasone (2 mg/kg), LPS plus Sal groups with different Sal doses (20, 40 mg/kg). Hemodynamic measurement and haematoxylin and eosin staining were performed. Serum levels of creatine kinase (CK), lactate dehydrogenase, the activities of the antioxidant enzymes catalase (CAT), superoxide dismutase (SOD), glutathione peroxidase (GSH‐px), glutathione, tumour necrosis factor‐α (TNF‐α), interleukin‐6 (IL‐6), and interleukin‐1β (IL‐1β) were measured after the rats were killed. iNOS, COX‐2, NF‐κB and PI3K/Akt/mTOR pathway proteins were detected by Western blot. *In vitro*, we evaluated the protective effect of Sal on rat embryonic heart‐derived myogenic cell line H9c2 induced by LPS. Reactive oxygen species (ROS) in H9c2 cells was measured by flow cytometry, and the activities of the antioxidant enzymes CAT, SOD, GSH‐px, glutathione‐S‐transferase, TNF‐α, IL‐6 and IL‐1β in cellular supernatant were measured. PI3K/Akt/mTOR signalling was examined by Western blot. As a result, Sal significantly attenuated the above indices. In addition, Sal exerts pronounced cardioprotective effect in rats subjected to LPS possibly through inhibiting the iNOS, COX‐2, NF‐κB and PI3K/Akt/mTOR pathway *in vivo*. Furthermore, the pharmacological effect of Sal associated with the ROS‐mediated PI3K/Akt/mTOR pathway was proved by the use of ROS scavenger, *N*‐acetyl‐l‐cysteine, in LPS‐stimulated H9C2 cells. Our results indicated that Sal could be a potential therapeutic agent for the treatment of cardiovascular disease.

## Introduction

Sepsis is a complex syndrome with multi‐organ dysfunction especially cardiovascular disease. Myocardial dysfunction and cardiac diastolic commonly occur in patients with severe sepsis. It should be noted that cardiovascular diseases remain a leading cause of morbidity and mortality around the world 1. As the most common source of cardiac injuries, myocardial infarction (MI) is characterized by pathological myocardial hypertrophy, heart failure, excessive generations of inflammatory cytokines and overproduction of reactive oxygen species (ROS) 2. ROS, the well‐known by‐products of normal cellular oxidative processes, can be produced under different stimuli including lipopolysaccharide (LPS). LPS is a main component of the outer membrane of Gram negative bacteria and has been used to induce cardiomyocytes lesion 3, 4.

The phosphoinositide 3‐kinase/protein kinase B/mammalian target of rapamycin mTOR (PI3K/Akt/mTOR) pathway is a critical cellular cascade in the cellular response to extracellular stimuli. Accumulating evidence indicated that PI3K/Akt/mTOR pathway participated in the cellular proliferation, differentiation, metabolism, cytoskeletal reorganization and apoptosis 5, 6. Additionally, previous investigator demonstrated that PI3K/Akt/mTOR cascade was driven by ROS 7.

Traditional Chinese medicine, widely used for centuries around the world, is still acknowledged as a main source of medicine 8, 9. *Rhodiola rosea* is a long‐standing herbal used to relieve high altitude sickness and protect erythrocytes against oxidative stress 10. As its important active ingredient, salidroside (Sal) (*p*‐hydroxyphenylethyl‐O‐β‐d‐glucopyranoside) has been reported to have various pharmacological properties, such as anti‐depressive 11, anti‐asthmatic 12, anti‐ulcer 13, neuroprotective 14, anti‐inflammatory 12 and antioxidative 15 effect. It was noteworthy that previous literatures proposed that Sal exhibited protective effect on myocardial injury 16. It was reported that Sal exerted remarkable benefits in inhibition of ROS overgeneration as an antioxidant in clinical patients. However, the mechanisms by which Sal scavenges ROS in LPS‐induced cardiac injury remain elusive 17. Thus, it was hypothesized that whether its antioxidative activity was attributed to the ROS‐mediated PI3K/Akt/mTOR signalling. The present study was designed to investigate the pharmacological effect of Sal on myocardial injury in LPS‐induced endotoxemic and explore the potential mechanism *via* the ROS‐mediated PI3K/Akt/mTOR pathway.

## Materials and methods

### Reagents

Sal (purity: 99%) was purchased from National Institutes for Food and Drug Control (Beijing, China). Lipopolysaccharides and *N*‐acetyl‐l‐cysteine (NAC) were provided by Sigma‐Aldrich (St. Louis, MO, USA). The ELISA kits for determinations of interleukin‐6 (IL‐6), IL‐1β and tumour necrosis factor‐α (TNF‐α) were produced by Nanjing KeyGEN Biotech. CO., Ltd (Nanjing, China). All biochemical kits were supplied by the Institute of Jiancheng Bioengineering (Nanjing, China). iNOS, COX‐2, p‐NF‐κBP65, NF‐κBP65, PI3K, P‐PI3k, P‐Akt, Akt, P‐mTOR and mTOR antibodies were purchased from Abcam (Cambridge, MA, USA).

### Cell culture and treatment

A rat cardiomyoblast H9C2 cell line, obtained from the Shanghai Institute of Biochemistry and Cell Biology (Shanghai, China), was cultured at 37°C under 5% CO_2_ in DMEM containing 10% foetal bovine serum (Hyclone, South America) with 100 IU/ml streptomycin and 100 IU/ml penicillin (Amresco, OH, USA). The H9C2 cells were passaged regularly and subcultured to 80% confluence before the experiments.

### MTT assay

The cells were seeded at 2 × 10^5^ cells/ml on 96‐well culture plates for 24 h. Then, various concentrations of Sal were added to the wells. After a 2‐h treatment, the cells were treated with LPS (4 μg/ml) for 24 h, 50 μl of MTT solution (5 mg/ml) (Sigma‐Aldrich) was added to each well and incubated at 37°C for 3 h. After that, each well was replaced by 150 μl DMSO and MTT‐formazan crystals were dissolved. Finally, the absorbance was measured at 570 nm using a microplate reader (Bio‐Rad Laboratories Ltd, Shanghai, China).

### Detection of intracellular reactive oxygen species generation

The cells were seeded at 2 × 10^5^ cells/ml on 96‐well culture plates for 24 h and then treated with various concentrations of Sal. Two hours later, the cells were stimulated with LPS (4 μg/ml). After 24‐h incubation, the cells were harvested for ROS detection. As the ROS scavenger, NAC (2 mM) was added prior to drugs. The concentration of NAC was chosen in according to the previous literature 18.

Intracellular ROS production was measured with a ROS assay kit (Beyotime, Nanjing, China). 2′,7′‐Dichlorofluorescein‐diacetate (DCFH‐DA) readily diffuses through the cell membrane and is enzymatically hydrolyzed by intracellular esterase to form non‐fluorescent DCFH, which is then rapidly oxidized to form highly fluorescent 2′,7′‐dichlorofluorescein (DCF) in the presence of ROS. The fluorescence intensity is proportional to ROS production. After incubated for 30 min, cells were washed with cold PBS three times and then the fluorescent intensity was assayed by the flow cytometry with excitation wavelength at 488 nm and emission wavelengths at 525 nm, respectively.

### Animal model

SD rats (200–250 g, 8‐week old) used in all experiments were obtained from Jiangning Qinglongshan Animal Cultivation Farm (Nanjing, China). The animals were housed in a constant temperature environment with a regular 12‐h light/dark cycle and provided with standard chow and water *ad libitum*. All the experimental procedures were carried out in accordance with the National Institutes of Health Guidelines for the Care and Use of Laboratory Animals.

**Figure 1:**
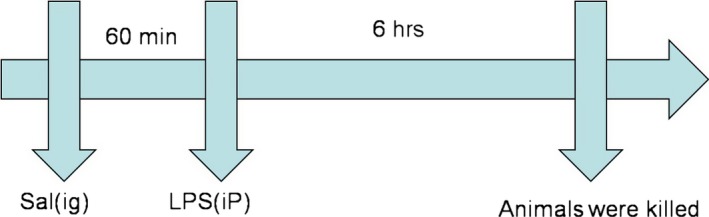
Animal treatment protocols in this study.

SD rats were randomly divided into five groups with 10 rats in each group: (1) control group, (2) LPS group, (3) LPS + dexamethasone (Dex, 2 mg/kg), (4) LPS + Sal (Sal, 20 mg/kg), (5) LPS + Sal (Sal, 40 mg/kg). The doses of Sal were chosen in accordance with the previous literatures 19, 20. Dex and Sal were given intragastrically for three consecutive days. Control group and LPS group were intragastrically treated with autoclaved PBS at the same volumes. One hour after the last administration, the rats in group (2, 3, 4, 5) were intraperitoneally challenged with 15 mg/kg LPS (*Escherichia coli* 055: B5). The control rats were intraperitoneally received autoclaved PBS. After 6‐h treatment of LPS, the animals were killed by Nembutal (sodium pentobarbital, i.p., 80 mg/kg/bodyweight, BW). A schematic diagram of the treatment schedule is shown in Fig. 1. The blood samples were collected by cardiac puncture for further analysis. Then the heart tissues were harvested with care, snap frozen in liquid nitrogen and stored at −80°C.

### Hemodynamic measurements

A standard limb lead II electrocardiogram was monitored continuously. The right carotid artery was cannulated with a polyethylene 90 catheter filled with heparin saline (500 U/ml) advanced to the lumen of the left ventricle. The cardiac LV function was evaluated by the left ventricular systolic pressure (LVSP), left ventricular end‐diastolic pressure (LVEDP), maximum LVP increase rate (LV + dp/dt_max_) and maximum LVP decrease rate (LV−dp/dt_max_) with a BL‐420s Biologic Function Experiment system (Chengdu, China).

### Determination of heart weight index

At the end of the experimental period, the rats’ BW was weighted and anaesthetized. Then the heart tissues (excluding large blood vessels and connective tissue) were immediately harvested and weighed after blotting with filter paper (heart weight, HW). The HW index (HWI) was computed as HWI = HW/BW.

### Activities of antioxidant enzymes in serum and cellular supernatant, CK and LDH in serum

The levels of CK, lactate dehydrogenase (LDH) and the activities of the antioxidant enzymes catalase (CAT), superoxide dismutase (SOD), glutathione peroxidase (GSH‐px) and glutathione (GSH) were determined according to the manufacturer's protocol 21.

### Cytokines in serum and cellular supernatant

Serum and cellular supernatant levels of IL‐6 and TNF‐α were measured by ELISA according to the manufacturer's instructions (R&D, Minneapolis, MN, USA). All measurements were performed in triplicate.

### Histological assessment

Immediately after the rats were killed, the hearts were excised and fixed in 10% formalin solution for 48 h. Then the heart tissue was processed for sectioning and staining by standard histological methods. Sections from the left ventricle were stained with haematoxylin and eosin and examined by light microscopy (Nikon, Tokyo, Japan).

### Western blotting

The cells were seeded at 2 × 10^5^ cells/ml on 96‐well culture plates for 24 h and then treated with various concentrations of Sal. Two hours later, the cells were stimulated with LPS (4 μg/ml). After 24‐h incubation, the cells were harvested for Western blot analysis. As the ROS scavenger, *N*‐acetyl‐l‐cysteine (NAC, 2 mM) was added prior to drugs.

The heart tissues and cells were homogenized, washed with PBS and lysed in a RIPA buffer (Beyotime). The protein concentrations were determined with a BCA protein assay (Beyotime). The samples were loaded on 10% sodium dodecyl sulphate polyacrylamide gels and were electrotransferred to nitrocellulose membranes. The membrane was blocked with 5% skim milk in Tris buffer and incubated with the appropriate specific antibodies. After washing, the blots were incubated with horseradish peroxidase‐conjugated second antibodies. The quantification of protein expression was normalized to GAPDH using a densitometer (Imaging System).

### Statistical analysis

All data were normally distributed and were expressed as mean ± SD. Results were analysed by one‐way anova with Tukey's multiple comparison test. *P* values <0.05 were considered to reflect a significant difference.

## Results

### Effect of Sal on MTT assay

**Figure 2:**
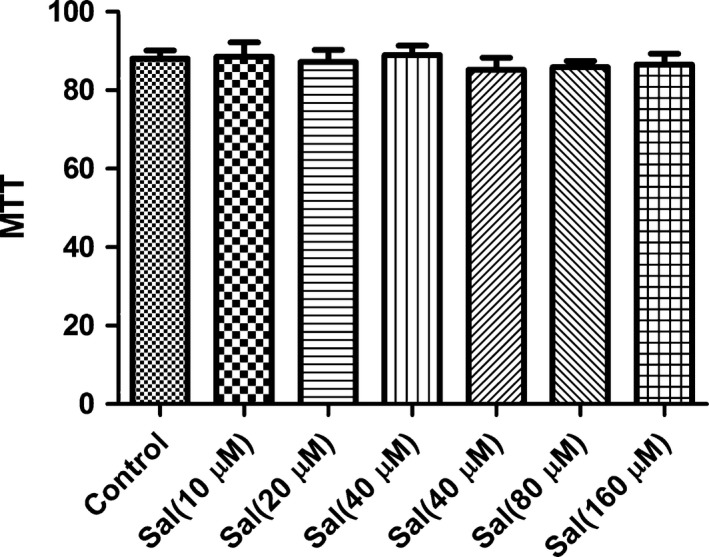
Effect of Sal on the viability H9c2 cells. Cells were cultured with Sal (10–160 μM) in the absence or presence of 4 μg/ml LPS for 24 h. Values are expressed as mean ± SD. Compared with control: ^##^ *P* < 0.01, ^###^ *P* < 0.001; compared with model: *P < 0.05, **P < 0.01,***P < 0.001.

To exclude the possibility that the pharmacological effect of Sal were because of its cytotoxity, we carried out MTT experiment after incubating with H9C2 cells. As expected, the concentrations of 10–40 μM Sal did not affect the cell viability in this study. Therefore, the inhibitory effect were not caused by the cytotoxicity of Sal (Fig. 2).

### Effect of Sal on ROS in LPS‐induced H9C2 cells

**Figure 3:**
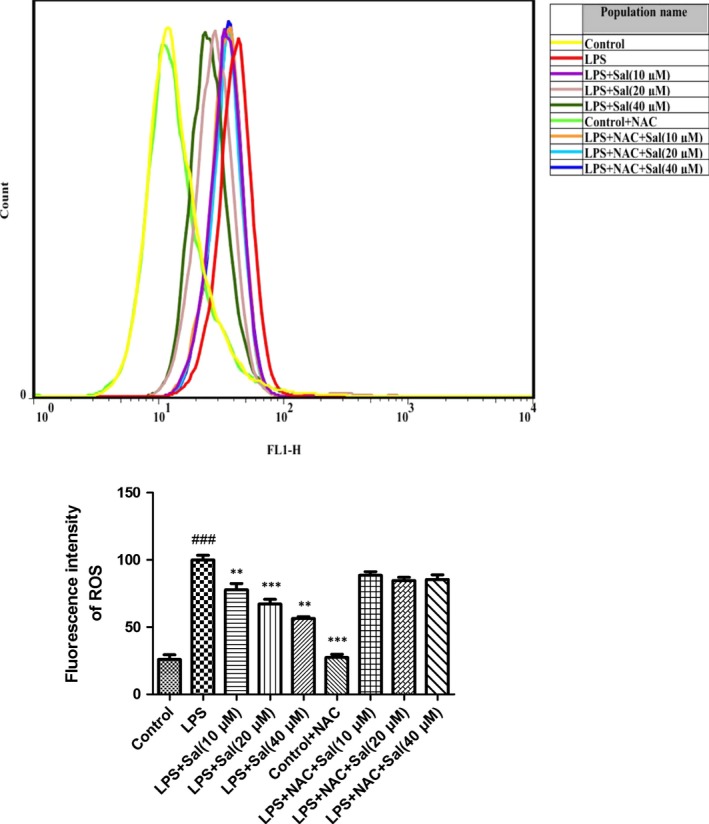
Effect of Sal on ROS in H9c2 cells. Values are expressed as mean ± SD. Compared with control: ^##^ *P* < 0.01, ^###^ *P* < 0.001; compared with model: *P < 0.05, **P < 0.01, ***P < 0.001.

To determine changes in the ROS level, we measured the oxidative conversion of the sensitive fluorescent probe DCFH‐DA to fluorescent DCF. The levels of ROS in H9c2 were pronouncedly increased after LPS administration. On the contrary, Sal effectively down‐regulated the ROS production in H9c2 cells in a concentration‐dependent manner (Fig. 3).

### Effect of Sal on LV function

**Figure 4:**
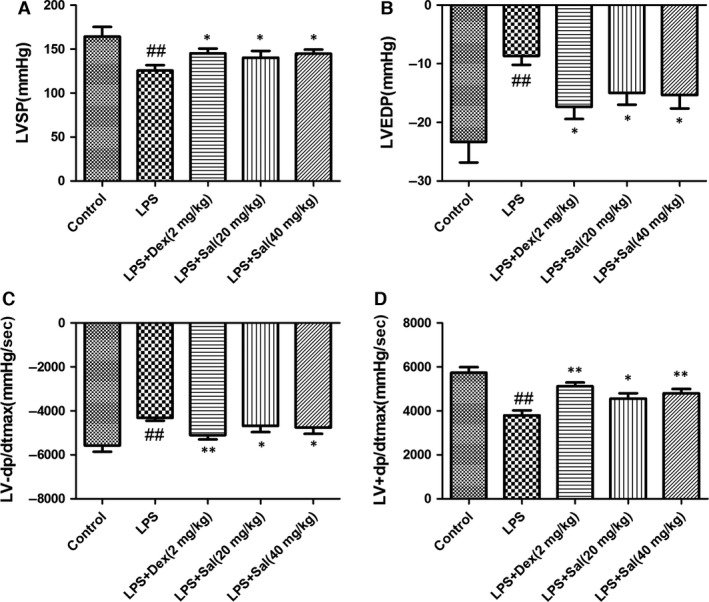
Effect of Sal on LV function indices including (**A**) left ventricular systolic pressure (LVSP), (**B**) left ventricular end‐diastolic pressure (LVEDP), (**C**) maximum LVP decrease rate (LV−dp/dt_max_) and (**D**) maximum LVP increase rate (LV+dp/dt_max_). Values are expressed as mean ± SD. Compared with control: ^##^ *P* < 0.01, ^###^ *P* <0.001; compared with model: *P < 0.05, **P < 0.01, ***P < 0.001.

Electrocardiographic patterns of control and experimental animals were depicted in Fig. 4. LVSP and LV + dp/dt_max_ in LPS group were notably reduced, whereas LVEDP and LV−dp/dt_max_ were increased compared with those in control group, which indicated that LPS challenge decreased LV function. On the contrary, these changes were considerably ameliorated by the Sal (20 mg/kg) and Sal (40 mg/kg) treatments. Our data indicated that Sal could attenuate the LV function in LPS‐induced myocardial injury.

### Effect of Sal on heart weight index (HWI)

**Figure 5:**
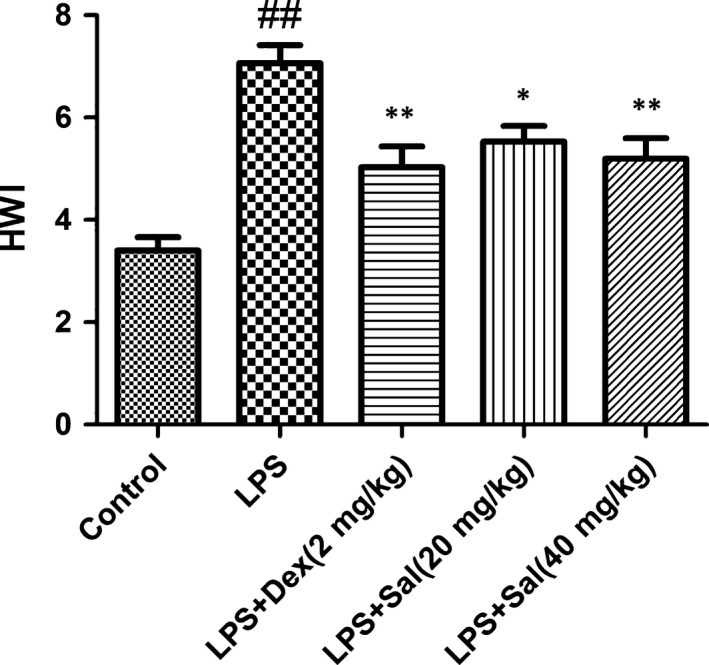
Effect of Sal on heart weight index. Heart weight index was calculated as HWI = heart weight (HW)/bodyweight (BW). Values are expressed as mean ± SD. Compared with control: ^##^ *P* < 0.01, ^###^ *P* < 0.001; compared with model: *P < 0.05, **P < 0.01, ***P < 0.001.

Heart weight index were greater in the LPS‐induced rats than that in the control group rats. With the pre‐treatment of Sal, HWI significantly decreased compared with that of LPS‐induced rats (Fig. 5).

### Effect of Sal on LDH in serum and cellular supernatant and CK in serum

**Figure 6:**
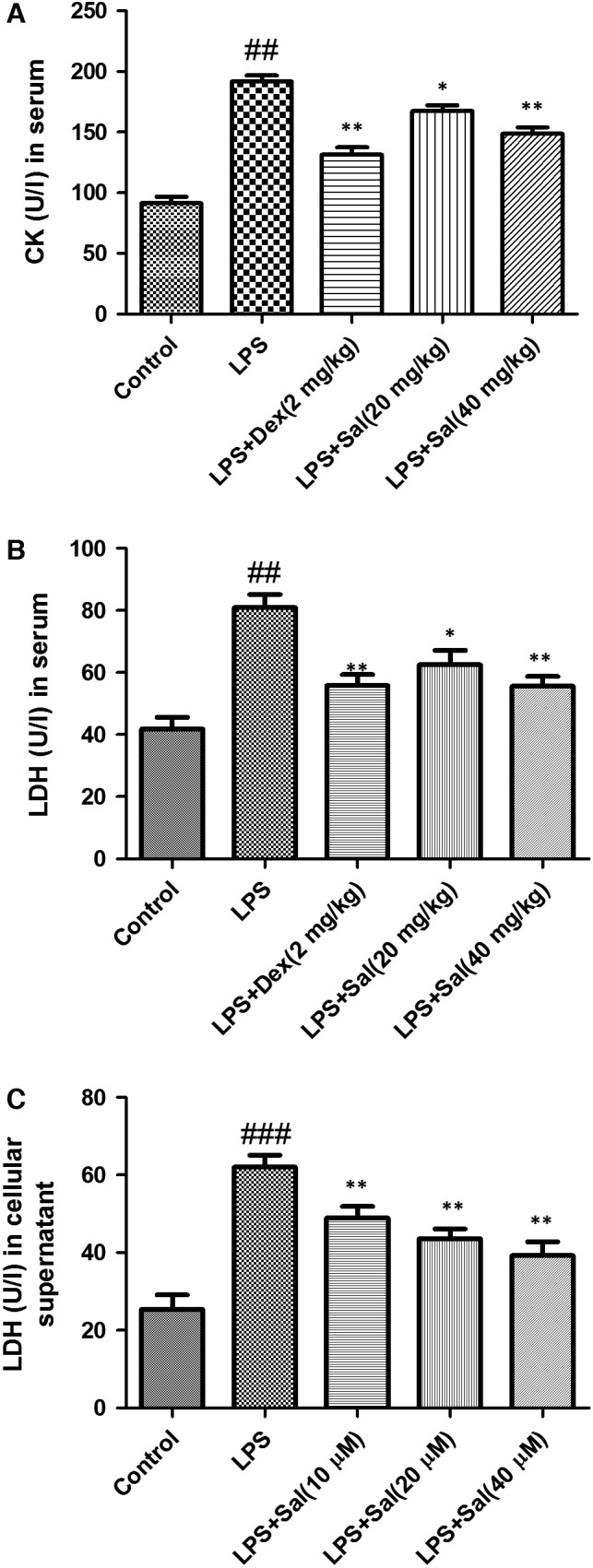
Effect of Sal on LDH, CK in serum and LDH in cellular supernatant. Values are expressed as mean ± SD. Compared with control: ^##^ *P* < 0.01, ^###^ *P* < 0.001; compared with model: *P < 0.05, **P < 0.01, ***P < 0.001.

To detect myocardial injury marker enzymes, the levels of CK‐MB and LDH were measured. The levels of LDH in serum and cellular supernatant were dramatically increased and the levels of CK in serum were significantly increased after LPS administration. Of note, treatment with Sal effectively decreased the levels of LDH, CK in serum and LDH activity in cellular supernatant (Fig. 6).

### Effect of Sal on activities of the antioxidant enzymes in serum and cellular supernatant

**Figure 7:**
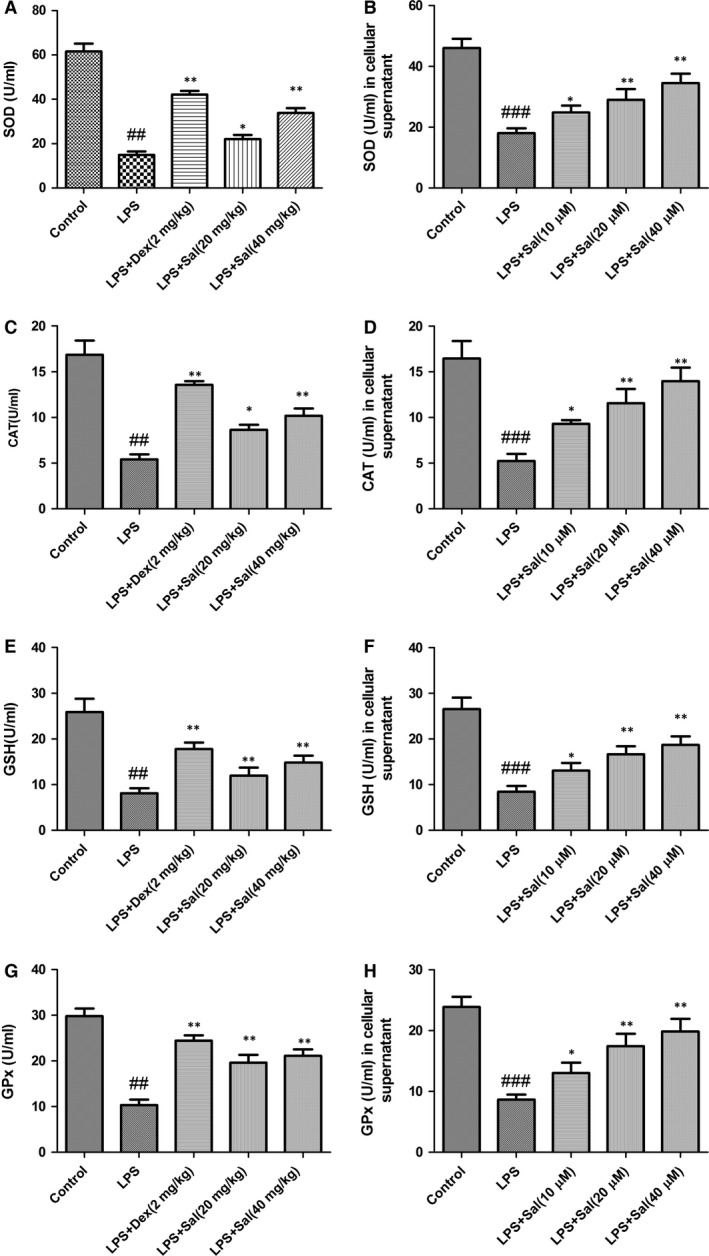
Effect of Sal on the levels of SOD, MDA, GSH, GPx in serum and cellular supernatant. Values are expressed as mean ± SD. Compared with control: ^##^ *P* < 0.01, ^###^ *P* < 0.001; compared with model: *P < 0.05, **P < 0.01, ***P < 0.001.

Lipid peroxidation in serum and cellular supernatant were determined by measuring the generations of CAT, SOD, GSH‐px and GSH. LPS stimulation significantly declined the SOD, CAT, GSH‐px, GSH activities and GSH content, respectively, while Sal treatment effectively restored these levels in serum and cellular supernatant. The analytical results suggested that Sal was capable of ameliorating oxidative stress in LPS‐stimulated rats (Fig. 7).

### Effect of Sal on inflammatory cytokines in serum and cellular supernatant

**Figure 8:**
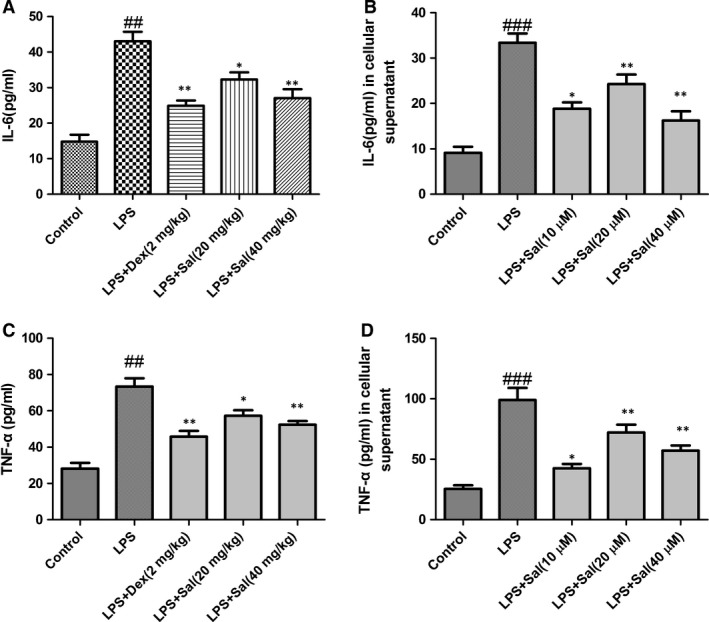
Effect of Sal on inflammatory cytokines in serum and cellular supernatant. Values are expressed as mean ± SD. Compared with control: ^##^ *P* < 0.01, ^###^ *P* < 0.001; compared with model: *P < 0.05, **P < 0.01, ***P < 0.001.

Next, we evaluated the generation of inflammatory cytokines in serum and cellular supernatant. The levels of the cytokines IL‐6 and TNF‐α in serum and cellular supernatant were dramatically increased after LPS administration. By contrast, Sal (20, 40 mg/kg) administration appeared to down‐regulate the IL‐6 and TNF‐α contents in dose‐dependent manners (Fig. 8).

### Effect of Sal on the expressions of iNOS, COX‐2 and NF‐κB in heart tissues

**Figure 9:**
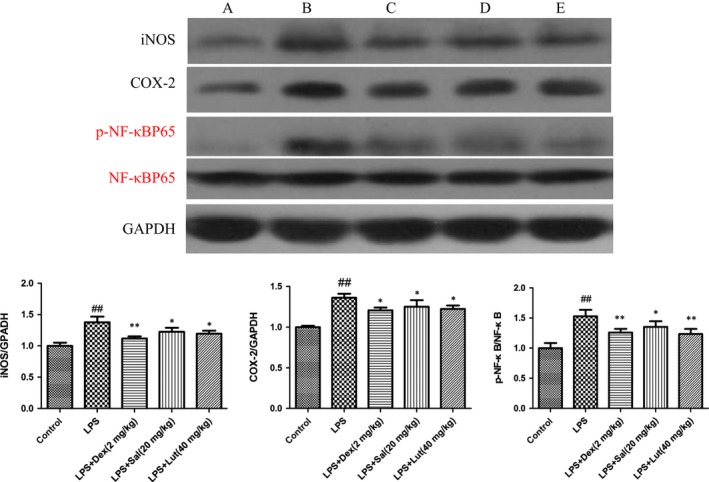
Effect of Sal on the protein expressions of iNOS, COX‐2 and NF‐κB in heart. Values are expressed as mean ± SD. Compared with control: ^##^ *P* < 0.01, ^###^ *P* < 0.001; compared with model: *P < 0.05, **P < 0.01, ***P < 0.001. **A**: Control; **B**: LPS;** C**: LPS + Dex (2 mg/kg); **D**: LPS + Sal (20 mg/kg); **E**: LPS + Sal (40 mg/kg).

To explore the potential mechanism of Sal on LPS‐induced myocardial injury, we investigated the protein expressions of iNOS, COX‐2 and NF‐κB in heart tissues. In response to LPS, the levels of iNOS, COX‐2 and p‐NF‐κB were evidently up‐regulated. As expected, Sal dramatically inhibited the expressions of iNOS, COX‐2 and phosphorylated NF‐κB. (Fig. 9).

### Effect of Sal on PI3K/Akt/mTOR in heart and H9c2 cells

**Figure 10:**
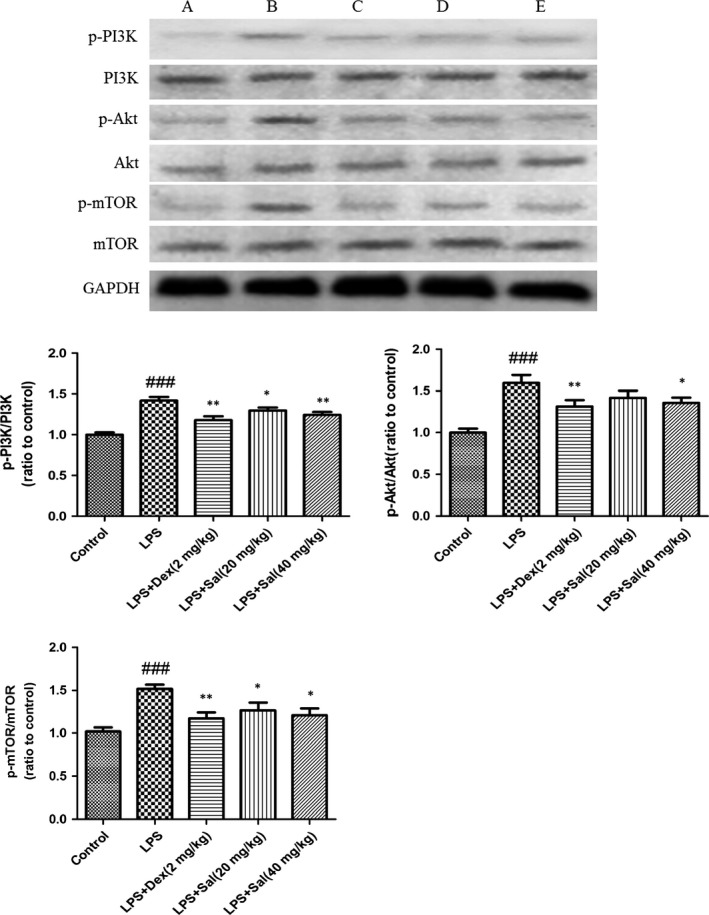
Effect of Sal on PI3K/Akt/mTOR in heart. Values are expressed as mean ± SD. Compared with control: ^##^ *P* <0.01, ^###^ *P* < 0.001; compared with model: *P < 0.05, **P < 0.01, ***P < 0.001. **A**: Control; **B**: LPS;** C**: LPS + Dex (2 mg/kg); **D**: LPS + Sal (20 mg/kg); **E**: LPS + Sal (40 mg/kg).

As illustrated in Fig. 10, the protein expressions of p‐PI3K, p‐Akt, p‐mTOR were dramatically up‐regulated after LPS stimulation. However, Sal significantly blocked the phosphorylations of the protein PI3K, Akt and mTOR in heart.

**Figure 11:**
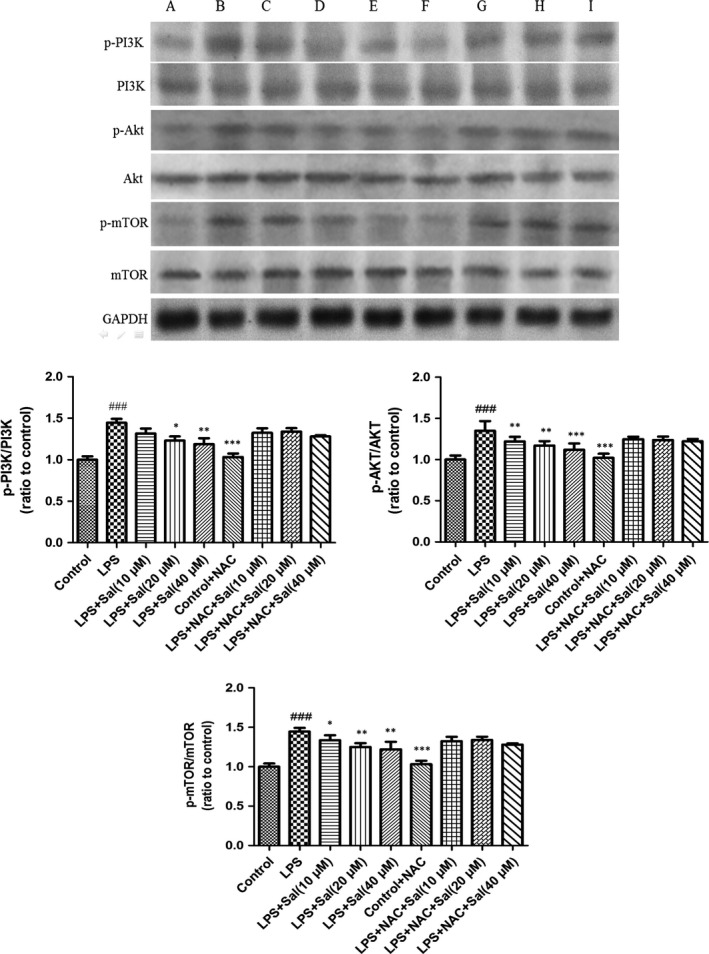
Effect of Sal on PI3K/Akt/mTOR in H9c2 cells. Values are expressed as mean ± SD. Compared with control: ^##^ *P* < 0.01, ^###^ *P* < 0.001; compared with model: *P < 0.05, **P < 0.01, ***P <0.001. **A**: Control; **B**: LPS;** C**: LPS + Sal (10 μM); **D**: LPS + Sal (20 μM); **E**: LPS + Sal (40 μM); **F**: Control + NAC;** G**: LPS + NAC + Sal (10 μM); **H**: LPS+ NAC + Sal (20 μM); **I**: LPS + NAC + Sal (40 μM).

To confirm the involvement of ROS in the execution of PI3K/Akt/mTOR pathway in LPS‐induced H9C2 cells, we further investigated the protein expressions of the above molecules. Notably, Sal effectively inhibited the up‐regulations of the phosphorylated PI3K, Akt and mTOR caused by LPS *in vitro*. Interestingly, after NAC incubation, the alterations were remarkably abrogated. These experimental data indicated the critical role of ROS in the regulation of PI3K/Akt/mTOR signalling (Fig. 11).

## Discussion

In the present study, we addressed that Sal had beneficial effect on LPS‐induced myocardial injury. Sal significantly attenuated the levels of LDH, CAT, SOD, GSH‐px, GSH, IL‐6, TNF‐α in serum and in cellular supernatant. Hemodynamic measurements and histopathological observations also confirmed the therapeutic effect of Sal. Western blot demonstrated that Sal inhibited the expressions of iNOS, COX‐2, NF‐κB and the phosphorylations of PI3K, Akt, mTOR in heart tissues. Additionally, we added the NAC, the inhibitor of ROS, to H9C2 cells; the *in vitro* experimental data suggested that the cardioprotective effect of Sal was possibly related to the ROS‐mediated PI3K/Akt/mTOR signalling.

Sal has been reported to have cellular protection through antioxidation 22 and the inhibition of free radicals 23. Although substantial researches revealed the beneficial role of Sal in myocardial ischaemia, there has been no literature to elucidate the underlying mechanism through PI3K/Akt/mTOR pathway in myocardial injury and try to explore the crucial role of ROS in this signalling cascade to date. This is the first study focused on the potential effect of Sal on MI *via* the ROS‐mediated PI3K/Akt/mTOR pathway in LPS‐induced rats and H9C2 cells.

Lipopolysaccharide is the component of the outer membrane of Gram negative bacteria 24 and is widely used as an inducer of endotoxemic in scientific studies. The entry of LPS into the lymphatic and circulatory systems brings about the systemic inflammatory response 25. Inflammatory process is highly related to various coronary diseases including acute MI, microvascular reperfusion injury and ischaemic heart disease myocardial necrosis 26. Some studies suggested that a substantial amount of TNF‐α is produced in cardiac myocytes exposed to LPS 27, 28. The pro‐inflammatory cytokines including TNF‐α and IL‐6 are involved in the initiation and regulation of inflammatory response 29. In our study, the overproductions of inflammatory cytokines induced by LPS were reflected by TNF‐α and IL‐6 elevation. However, Sal treatment significantly suppressed the contents of TNF‐α and IL‐6 in both serum and cellular supernatant, which suggested that its cardioprotective effect were possibly associated with anti‐inflammatory properties.

LDH, the specificity myocardial enzymes in the cytoplasm, releases into blood during myocardial ischaemia. Additionally, CK‐MB distributes in the myocardium and is widely considered as the diagnostic index for myocardial membrane 30. These two kinds of enzymes are often acknowledged as the criteria in myocardial ischaemia injury. The data showed that the treatment with Sal inhibited the activities of these enzymes, which proved that ES exhibited the potential cardioprotective effect. Several pieces of evidence revealed that free radicals contribute to the loss of membrane integrity and the peroxidation of lipid membranes 31. Thus, the various myocardial enzymes released from the damaged tissue to the circulation 32. SOD exerts multifaceted physiological activities including anti‐inflammatory and antioxidant effect 33. In addition, malondialdehyde (MDA) is the end‐product of polyunsaturated fatty acid and usually used as an indicator for evaluating the lipid peroxidation in heart 34. CAT and GSH are crucial non‐protein antioxidants and scavenge the lipid peroxide radicals. GSH‐px cleans up oxidative product and is associated with the transportation of GSH. The amelioration of lipid peroxidation may be the effective pharmacological intervention for treating myocardial ischaemia 35. Our present data showed that the treatment with Sal significantly reduced the content of MDA, restored SOD, CAT, GSH and GSH‐px activity. These analytical results suggested that the cardioprotective effect of Sal might be attributed to its antioxidative activity. COX‐2 and iNOS are crucial enzymes involved in the progression of inflammatory and oxidative dysfunction 36. NF‐κB is an essential transcriptional factor modulating inflammatory mediators and driving the expressions of iNOS and COX‐2 37. Evidence has emerged indicating that the iNOS and COX‐2 which regulated by NF‐κB are closely associated with the LPS‐induced myocardial disorder 38. Our experimental results suggested that Sal effectively inhibited the up‐regulations of iNOS, COX‐2 and p‐NF‐κB in LPS‐induced myocardial injury.

ROS, including superoxide (O^2−^), hydroxyl radical (HO^−^) and hydrogen peroxide (H_2_O_2_), play paradoxical roles in cellular environments 39. Appropriate levels of ROS assist in mounting an effective defence against pathogens. However, sustained overproduction of ROS is believed to conduce to cellular damage 40.

**Figure 12:**
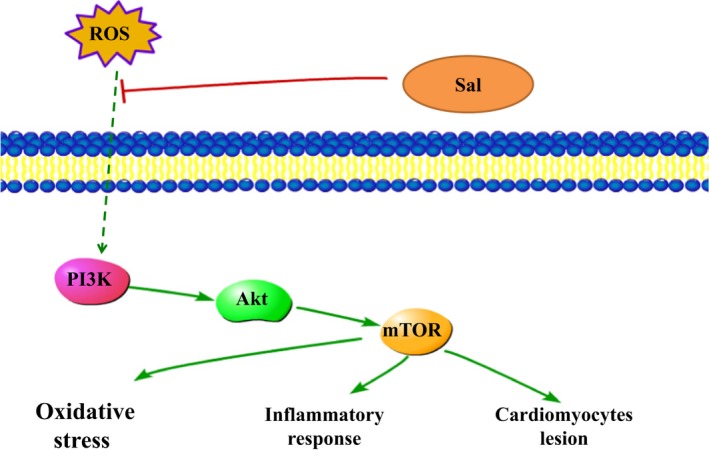
Pathways of Sal in LPS‐induced H9C2 cells.

The serine/threonine kinase mTOR belongs to the PI3K‐related kinase (PIKK) family and is reported to be related to ROS 41. PI3K triggers Akt which consequently activates mTOR. In mammals, mTOR is governed by a kinase cascade involving PI3K and Akt 42. The stimulation of PI3K/Akt‐dependent cascade may decrease the morbidity and mortality caused by MI 43. Previous investigator indicated that PI3K/Akt pathway mediated the cardioprotection in ischaemia‐induced myocardial apoptosis with the application of PI3K inhibitor LY294002 44. The overexpression of mTOR attenuates the inflammatory response and inhibits cardiac fibrosis in myocardial I/R lesion 45. Chronic activation of Akt/mTOR signalling can contribute to pathological cardiac hypertrophy 46. Wang *et al*. also demonstrated that the p‐Akt and p‐mTOR were significantly changed in myocardial ischaemia/reperfusion injury 47. Herein, our data implied that Sal resulted in the ameliorations of PI3K/Akt/mTOR expressions in LPS‐induced MI *in vivo* and *in vitro*. The administration of NAC, the inhibitor ROS, could rescue the phosphorylations of the above proteins. Moreover, the expressions of the phosphorylated and non‐phosphorylated PI3K/Akt/mTOR signalling pathways in the NAC‐treated groups showed significant alterations compared with those in the NAC untreated groups, which indicated that ROS acted as an upstream event of PI3K, Akt and mTOR. The analytical results suggested that Sal exhibited protective effect on LPS‐induced MI through ROS‐mediated PI3K/AKT/mTOR signalling pathway. Simplified overview of the above signalling pathways was as illustrated in Fig. 12.

In conclusion, the present study demonstrated that the Sal administration improved cardiac function after LPS‐stimulated endotoxemic in rats. The cardioprotective effect of Sal might be attributed to its ability of suppressing myocardial lipid peroxidation and inhibiting inflammatory cytokines both *in vivo* and *in vitro*, possibly through the inhibition of the ROS‐mediated PI3K/AKT/mTOR pathway. Therefore, our results indicated that Sal could be a potential therapeutic agent for the treatment of cardiovascular disease. Further studies are warranted to explore the clinical application of Sal.

## Competing interest

The authors declare that they have no competing interests.

## Author contribution

Li Chen and Peng Liu, Xin Feng, Chunhua Ma conceived of the study, carried out the work, participated in its design and coordination. Chunhua Ma and Lvyi Chen participated in the design of the study and financially supported the study. All authors read and approved the final manuscript.
